# Developing research priorities for palliative care of people with intellectual disabilities in Europe: a consultation process using nominal group technique

**DOI:** 10.1186/s12904-016-0108-5

**Published:** 2016-03-24

**Authors:** I. Tuffrey-Wijne, M. Wicki, P. Heslop, M. McCarron, S. Todd, D. Oliver, A. de Veer, G. Ahlström, S. Schäper, G. Hynes, J. O’Farrell, J. Adler, F. Riese, L. Curfs

**Affiliations:** Kingston University & St George’s University of London, Faculty of Health, Social Care and Education, Cranmer Terrace, London, SW17 0RE UK; Internationale Hochschule für Heilpädagogik Zürich, Zürich, Switzerland; Norah Fry Research Centre, School for Policy Studies, University of Bristol, Bristol, UK; Trinity College, University of Dublin, Dublin, Ireland; Faculty of Life Sciences and Education, University of South Wales, Cardiff, Newport, UK; Tizard Centre, University of Kent, Kent, UK; Netherlands Institute for Health Services Research, Utrecht, The Netherlands; Lunds Universitet, Lund, Sweden; Department Muenster, Catholic University of Applied Sciences, Muenster, Germany; University of Applied Sciences of Special Needs Education, Zürich, Switzerland; Division of Psychiatry Research and Psychogeriatric Medicine, Zurich, Switzerland; Maastricht University Medical Centre, Governor Kremers Centre, Maastricht, The Netherlands

**Keywords:** Intellectual disabilities, Palliative care, Research priorities, Nominal group technique, Consensus methods, Health services research

## Abstract

**Background:**

Empirical knowledge around palliative care provision and needs of people with intellectual disabilities is extremely limited, as is the availability of research resources, including expertise and funding. This paper describes a consultation process that sought to develop an agenda for research priorities for palliative care of people with intellectual disabilities in Europe.

**Methods:**

A two-day workshop was convened, attended by 16 academics and clinicians in the field of palliative care and intellectual disability from six European countries. The first day consisted of round-table presentations and discussions about the current state of the art, research challenges and knowledge gaps. The second day was focused on developing consensus research priorities with 12 of the workshop participants using nominal group technique, a structured method which involved generating a list of research priorities and ranking them in order of importance.

**Results:**

A total of 40 research priorities were proposed and collapsed into eleven research themes. The four most important research themes were: investigating issues around end of life decision making; mapping the scale and scope of the issue; investigating the quality of palliative care for people with intellectual disabilities, including the challenges in achieving best practice; and developing outcome measures and instruments for palliative care of people with intellectual disabilities.

**Conclusions:**

The proposal of four major priority areas and a range of minor themes for future research in intellectual disability, death, dying and palliative care will help researchers to focus limited resources and research expertise on areas where it is most needed and support the building of collaborations. The next steps are to cross-validate these research priorities with people with intellectual disabilities, carers, clinicians, researchers and other stakeholders across Europe; to validate them with local and national policy makers to determine how they could best be incorporated in policy and programmes; and to translate them into actual research studies by setting up European collaborations for specific studies that require such collaboration, develop research proposals and attract research funding.

## Background

### Intellectual disabilities and palliative care

There have been mounting concerns in recent years about inequalities in the provision of healthcare for people with intellectual disabilities [1–5]. People with intellectual disabilities are more likely to have poorer health outcomes than the general population, for reasons including poor access to healthcare services [3], poor quality healthcare [6], lack of advance care planning [6], lack of staff understanding of their needs [7–9], and diagnostic overshadowing (where the symptoms of physical ill-health are attributed to the presence of intellectual disability, and therefore not treated or managed [10]).

Intellectual disability affects approximately 1–3% of the population [11]. The term covers a wide range of abilities and disabilities, skills and limitations, but always includes the following three aspects: (a) a significantly reduced ability to understand new or complex information and to learn and apply new skills (impaired intelligence); (b) a significantly reduced ability to cope independently, expressed in conceptual, social, and practical adaptive skills (impaired adaptive functioning); and (c) early onset (before adulthood), with a lasting effect on development [12, 13].

The specific palliative care needs of people with intellectual disabilities have only recently become a focus of research attention. Early work in this field was concerned with the impact of bereavement on people with intellectual disabilities [14]. The earliest reported research within the English-language literature related to intellectual disability, death and dying date from the 1980s and looked at patterns of morbidity and mortality within institutionalised populations [15]. The first large scale mortality studies that compared data of people with intellectual disabilities with those of the general population came at the turn of the century from Finland [16] and Ireland [17], where national registers of the population of people with intellectual disabilities made this possible. Countries without such registers face significant methodological difficulties in making such comparisons [18, 19]. Most such studies reported earlier deaths among people with intellectual disabilities.

The first case reports of adults with intellectual disabilities who were dying and in need of palliative care were published in the 1990s [20, 21], but research studies in this field did not appear until this century. A literature review in 2007 revealed that of the 45 identified journal papers, book chapters and web-based articles with a primary focus on palliative care issues for people with intellectual disabilities, only seven of the materials were research reports [22]. Although research activity in this area has been growing steadily in the past decade, there is still only limited empirical evidence about applying the principles of palliative care to people with intellectual disabilities who have palliative care needs [23]. It has been argued that the lack of research interest in death, dying and intellectual disability stems from concerns that it is too emotive, as well as from the fact that the lives and deaths of people with intellectual disabilities have been hidden from society until the time of deinstitutionalisation [24, 25].

The palliative care needs of people with intellectual disabilities may be no different from those of the rest of the population, but they often present with unique challenges and circumstances that make it more difficult to meet those needs. A recent review of both USA and European literature found that people with intellectual disabilities face unique barriers in accessing hospice care, including delays in diagnosis of life-limiting conditions, the hospice providers’ knowledge of this patient group, issues around communication, and ethical dilemmas including issues around informed consent [26]. Difficulties with communication and comprehension affect the assessment and treatment of pain and other symptoms [27, 28]. The complex issues around ethics, decision making and patient involvement have become a more recent focus for research [29–31]. There is evidence that care at the end of life is of poorer quality for people with intellectual disabilities compared with the general population [32].

A significant proportion of the research studies to date is focused on the experiences of staff providing palliative care to people with intellectual disabilities, highlighting the training needs of palliative care professionals who may have limited experience of providing care for this population and the importance of collaboration between intellectual disability services and palliative care services [8, 26, 33–36].

There are a number of challenges in researching the palliative care needs of people with intellectual disabilities. One of the difficulties, especially in an international context, is the lack of consensus on terminology and variation in service models of palliative care [37, 38] and intellectual disability [39]. In some countries, for example, the definition of intellectual disabilities may or may not include people with mild intellectual disabilities or people on the autistic spectrum who don’t have additional intellectual disabilities. There are various ways in which the population of people with intellectual disabilities might be identified, such as through access to specialist services or medical records; each have their limitations. Some countries have highly developed palliative care services; in others, no such services are available even to the general population [40]. This makes it difficult to make international comparisons.

In order to understand the issues involved, much of the research has used samples of services, staff and carers for interviews or surveys. Investigating the perspectives of people with intellectual disabilities themselves presents significant methodological and practical difficulties [41], although these are not unsurmountable [42, 43]. Another difficulty has been the lack of understanding and agreement on what constitutes good palliative care for people with intellectual disabilities and a lack of common outcome measures for assessing this. Finally, in assessing the current state of knowledge on an international level, there is clearly a limitation in only accessing English-language literature, which is likely to skew insights towards those of English-speaking countries.

### Developing research priorities

Setting a research agenda for palliative care of people with intellectual disabilities is important for a number of reasons. Firstly, it addresses a gap in national strategies for palliative care and research, where there appears to be a distinct lack of focus on the needs of people with intellectual disabilities. There are research gaps for the general population too, but people with intellectual disabilities are often explicitly excluded from research populations, including palliative care research. Secondly, obtaining a consensus on the most important areas for research ensures that existing research expertise in this area is well used and may encourage researchers new to these topic areas to help expand the knowledge base. Thirdly, it informs and encourages international collaboration, and stimulates mutual sharing of expertise. This is particularly important in a European context where research and clinical expertise are unevenly distributed, with most developments originating in North West Europe. International collaboration in research has a wide range of benefits, including knowledge transfer, developing culturally relevant palliative care services, facilitating successful funding applications and establishing networking mechanisms that enable multi-country comparative studies [44]. Finally, identifying consensus research priorities may improve the possibilities of attracting funding in areas where it is most needed.

For over a decade, researchers in this field have shared their findings at dedicated “Death and Dying” symposia during international conferences, in particular through the European Association of Palliative Care (EAPC) and the International Association for the Scientific Study of Intellectual and Developmental Disabilities (IASSIDD). There have been several international research collaborations. In recognition of the barriers people with intellectual disabilities face in accessing palliative care services [45], the EAPC convened a Taskforce on Intellectual Disabilities in 2012, consisting of clinical and academic experts in the field. The taskforce developed consensus norms for palliative care of people with intellectual disabilities in Europe, which has been published as a White Paper [46]. Following this, a group of 16 researchers and clinicians in the field of palliative care for people with intellectual disabilities gathered for round-table workshops to share experiences and knowledge, assess the existing body of research, identify the challenges in conducting research in this field and strengthen international collaboration and networks. The meeting was convened in response to a desire by European researchers to develop joint projects. Part of this process was to work towards proposing a consensus research agenda. This paper reports on the outcome of one of the workshops, focusing on the following question that was posed: “What are the research priorities for palliative care of people with intellectual disabilities in Europe?” There are clearly limitations related to the small size of the project. However, the authors believe that it is important to share work of this kind in order to help researchers, policy makers and practitioners direct their focus towards some of the most pertinent issues. Resources in this area are scarce and need to be well-used.

## Methods

The participants were academics and clinicians who had been involved in research projects related to aspects of palliative care for people with intellectual disabilities within Europe. The group was convened through networking and included members of the EAPC Taskforce on Intellectual Disabilities as well as participants and presenters of scientific papers at the round table “Palliative Care for People with Intellectual Disabilities in Europe” and the “Death & Dying” symposium of the European congress of the International Association for the Scientific Study of Intellectual and Developmental Disabilities (IASSIDD) in Austria (July 2014).

Discussions were held over two days in February 2015 at the University of Applied Sciences of Special Needs Education in Zürich, Switzerland.

The first day involved round table presentations of the participants’ research to date, followed by discussions about perceived gaps in knowledge and the methodological, practical and ethical issues of collecting data around death, dying and palliative care for people with intellectual disabilities. The second day focused on the identification of research priorities using nominal group technique methodology.

Although well-established research methodology (nominal group technique) was used, this was a consultation process involving research professionals and clinicians, rather than an empirical study.

Due to its methodology, the process did not fall under the Swiss Federal Act on Research on Human Beings and was therefore not subject to ethics review by the cantonal ethics committee in Zurich (where the workshop was held). All participants provided consent for study participation and publication or their views.

### Using nominal group technique (NGT) to build consensus

NGT [47] uses a democratic process to form a set of collective priorities. The highly structured technique allows for the sharing of ideas from groups of people who have insight into a specific area of interest, which are then discussed and ranked by the group, producing consensus decisions [48]. NGT methodology has been used successfully to determine research priorities in healthcare [49–53], including palliative care in under-researched areas [44]. Within palliative care settings, it has also been used to identify outcome indicators [54], patient and carer preferences for end of life care [55–57], and staff perspectives on the information needs of family caregivers [58].

NGT has a number of advantages over other group methods. It prevents domination by more vocal or powerful members of the group and avoids conformity or social pressure [59, 60]. It is time and money efficient, as it is a single-occasion process that uses few resources to acquire a substantial amount of information in a relatively short time [48]. It requires little preparation by the participants and allows for immediate dissemination of results to the group, which makes it a highly satisfying method for the participants [48].

NGT in this project lasted approximately two hours and was facilitated by the first author, who was experienced in using the methodology [55]. Following explanation of the process, the NGT session was conducted in five steps.

Step 1: *Silent generation of research priorities.* Participants were asked to take 15 min to consider the following question: “What are the research priorities for palliative care of people with intellectual disabilities in Europe?” and write down, individually, as many answers as they wanted. To help with this they were given a printed summary of the previous day’s discussions, prepared by the second author.
Step 2: *‘Round robin’ recording of research priorities.* Each participant in turn was asked to name one research priority at a time, which was recorded on a flip chart. No discussions, questions or comments were allowed at this stage. Participants were instructed to contribute only those research priorities that had not already been mentioned, or that added a different perspective on ideas already on the flip chart; if they had no new ideas, they could pass. Three rounds were held, after which the participants were asked collectively whether there were any further new ideas.
Step 3: *Clarification of research priorities.* The facilitator went through all the listed research priorities to ensure that everyone understood what was meant by them. When needed, participants could ask questions and offer clarifications.
Step 4: *Collapsing of research priorities.* The facilitator and several participants organised all the listed research priorities into groups, simplifying them and bringing together similar ones in thematic categories. These were written on a new flip chart and discussed with the group, in order to ensure that all participants understood and approved of the congregated research themes.
Step 5: *Ranking of research priorities.* Each participant was asked, individually and anonymously, to select the five most important research themes and rank them in order of importance.

Each participant allocated between one and five points to their five selected research themes, with their first preference receiving five points and their fifth preference receiving one point. All scores were added together, resulting in a group ranking of priorities. The number of votes each theme received was also noted, as this indicated not just the strength but the breadth of support for the theme.

## Results

Sixteen people from six European countries took part in the first day of the process, sharing nine presentations of past, current and planned research projects in the area of palliative care for people with intellectual disabilities. Twelve people took part in the NGT process the following day; the others were unable to attend both days (see Acknowledgments for details of participants).

At the outset of the NGT process on the second day of the workshop, 40 research priorities were proposed. They were collapsed into eleven research themes. Three of these emerged as major research themes, covering nine, eight and seven of the proposed research priorities respectively. The other eight themes each covered one, two or three of the proposed research priorities (see Table 1).

**Table 1:** “Round robin” results: identified research priorities palliative care (PC) of people with intellectual disabilities (ID)

Not in order of importance; grouped in categories for ease of reading	
Mapping	
1. Mapping PC services accessed by people with ID in Europe (availability; usability)	
2. Collect data on death and dying across Europe in the general population and inclusion of people with ID in those data *(Clarification: investigate existing data bases)*	
3. Mapping transitions of people with ID at EoL, incl impact on the patient	
4. How many people with ID are currently on PC pathways? How are they identified, what are their characteristics; at local/national/international level? *(Clarification: what is the scale, scope and cost of the problem?)*	
5. What healthcare services do people with ID access at EoL across Europe?	
6. What are the relevant (inter) national laws across Europe? *(Clarification: laws affecting the PC of people with ID)*	
7. What is the provision of care for people with ID dying of cancer?	
8. What are the societal attitudes towards dying and people with ID across Europe?	
Quality of care	
1. Level of patient involvement in all aspects of PC (actual and preferred)	
2. Develop a bank of case studies on organisation development, to develop PC for people with ID in one research framework	
3. What is the quality of care provided to people with ID in Europe? What explains variability?	
4. What are the challenges in achieving the Consensus Norms across Europe?	
5. What are best practice norms for PC for the general population and for people with ID across Europe? *(Clarification: What are the national indicators for good PC practice?)*	
6. What are the perspectives on PC needs from: people with ID; family/carers; staff – across Europe?	
7. Identifying facilitators and barriers to achieving high quality PC for people with ID throughout Europe	
8. What are the experiences of families and (paid) carers across Europe?	
9. Understanding best practice models for advanced dementia	
End of life decisions	
1. Develop a decision making framework for EoL care decisions (to support staff/family)	
2. What are the methods of participation in EoL decisions for people with severe/profound ID across Europe?	
3. How to communicate with people with ID about their situation/illness in order to facilitate their involvement in care/decisions?	
4. Factors determining prevalence and nature of EoL decisions	
5. How do we develop Advance Care Planning for people with ID? (How? When? Who? etc.)	
6. What is the process for deciding a person with ID needs PC? How is that communicated?	
7. Does withholding/withdrawing treatment, and assisted dying, differ for people with ID and the general population? What is the effect on people with ID, carers, professionals?	
Strategic/policy	
1. What is the influence of (inter) national policies and guidelines on PC provision for people with ID? How can policies be used to improve provision?	
2. How to connect individual Person Centred Planning procedures with social care/healthcare/ political planning?	
Training	
1. Develop training programme; implement and evaluate	
2. Develop learning programmes on death and dying for people with ID	
3. Developing and collating (inter) national resources: training, information materials	
Guidelines and tools on individual level	
1. What is the impact of breaking bad news on people with ID?	
2. How do we assess symptoms and PC needs of people with ID?	
Outcome measures	
1. Identifying and testing patient related outcome measures for PC for people with ID across Europe (i.e. a common instrument); compare and contrast with general population	
2. Test existing instruments / develop new instruments to identify PC needs and priorities for people with ID across Europe and across services	
Collaborative working	
1. How to promote collaborative working in settings across Europe?	
2. Test ways to improve PC for people with ID in a sample of European regions/countries	
Economics	
1. What maximises good results for PC for people with ID at EoL, in the most cost-effective manner?	
2. How to develop economic models for PC for people with ID?	
Definitions and philosophies	
1. Clarify and agree definitions: PC, ID, Europe	
2. Investigate and critique the philosophy of PC from an ID perspective	
Review	
1. Analyse/review work already done in this area	

The themes were summarised and presented to the participants, each of whom ranked them in order of importance (see Table 2).

**Table 2:** Summarised research priorities, ranked in order of importance

Ranking	Research priority	1^st^ choice *5 points*	2nd choice *4 points*	3rd choice *3 points*	4^th^ choice *2 points*	5^th^ choice *1 point*	Total score *(Number of votes)*
1	Investigating issues around end of life decision making. *This could include:*	3	2	3	2	2	38 *(12)*
	• Profile of end of life decisions for people with ID, incl: starting PC pathways, withholding/withdrawing treatment						
	• Methods and processes of decision making						
	• Influencing factors on decision making						
	• Patient participation in decision making, incl communication issues						
	• Developing a decision making framework						
2	Mapping the scale and scope of the issue: *What is the current state of affairs with regards to PC for people with ID, and how does this compare to the general population? This could include:*	4	3	-	1	-	34 *(8)*
	• Access to healthcare and PC services						
	• Societal attitudes towards dying and people with ID						
	• Relevant national and international laws						
	• People with ID, cancer and access to cancer services						
	• Transitions between services						
3	Investigating the quality of PC for people with ID.	3	2	2	1	1	32 *(9)*
	*This could include:*						
	• Challenges in achieving best practice (Consensus Norms)						
	• Perspectives and experiences of people with ID, families, carers, staff						
	• Levels of patient involvement						
	• Understanding best practice models for dementia						
	• Collating case studies on patients and/or organisational developments						
4	Developing outcome measures and instruments for PC of people with ID. *This could include:*	2	1	1	2	1	22 *(7)*
	• Common European instrument for measuring quality of PC for people with ID						
	• Adapting existing measures (incl contrast with general population)						
	• Developing new measures/instruments						
5	Clarifying definitions and philosophies:	-	2	1	-	2	13 *(5)*
	*Develop a common language*						
	“Intellectual disabilities”, “Palliative care”, “Europe”						
6	Developing specific tools and guidelines to improve PC of individuals with ID *(and investigating the impact of such tools). This could include:*	-	1	1	2	1	12 *(5)*
	• Assessment of pain and other symptoms						
	• Breaking bad news						
7	Focusing on training and resources.	-	1	-	2	1	9 *(4)*
	• Develop resources, incl. training programmes						
	• Collate (inter) national resources (training, information)						
8	Investigating economic issues. *This could include:*	-	-	1	-	3	8 *(4)*
	• *Developing economic models*						
	• *Investigating ways to maximise good results in cost-effective ways*						
9	Promoting collaborative working. *This could include:*	-	-	1	-	2	5 *(3)*
	• *Testing ways to improve care through collaboration*						
10	Investigating policies and strategies. *This could include:*	-	-	1	1	-	5 *(2)*
	• *Investigating connections between local needs/person-centred*						
	• *Influence of (inter) national policies on PC for people with ID*						
	• *Plans with national policies and service provision*						
11	Review and analyse work already done.	-	-	1	-	-	3 *(1)*

Four major research themes received the highest rankings. The top priority was “Investigating issues about end of life decision making”, which was the only research theme ranked in the top five by all 12 participants. The second priority was “Mapping the scale and scope of the issue”. The third priority was “Investigating the quality of palliative care for people with intellectual disabilities”, including the challenges in achieving best practice. The fourth priority was “Developing outcome measures and instruments for palliative care of people with intellectual disabilities”.

Following immediate feedback of the NGT results to participants, the group was able to focus their discussions on these four main research priorities and started to consider an action plan for future research planning and collaborations.

## Discussion

Undertaking research is important for identifying problems and testing solutions. In order to optimise limited research capacity, a group of researchers and practitioners sought to develop research priorities for the palliative care of people with intellectual disabilities. However, the results reported here should be seen as a starting point for further discussion and validation, rather than an end product. A clear limitation of the approach was the relatively small group size; a different group of participants may have resulted in a different ranking of priorities. Participants’ responses may have been given in line with their own personal research agendas and priorities. Furthermore, whilst the participants in the workshop were experts who had gained national and international recognition for their work in the field, a number of other equally experienced researchers were unable to attend.

More importantly, the process did not include people with intellectual disabilities themselves or their families. Whilst the opinions of the researchers and clinicians involved are likely to be valuable, we acknowledge the importance of accessing the opinions and priorities of other stakeholders (including people with intellectual disabilities themselves) both in setting research priorities and in being involved in the design and conduct of research studies. Cross-validation of the proposed priorities with other stakeholder groups is therefore an important next step.

A further limitation of the approach was that the majority of participants came from northern European countries where there are integrated palliative care services [40]; none attended from eastern or southern countries of Europe so the identified research priorities may not be representative of the whole of Europe. Despite these limitations, the workshop represented a gathering of research teams and individual researchers who were not all from English-speaking countries; most had not previously collaborated on palliative care research in ID.

The top four research themes (see Table 2) identified in the workshops represent important areas for future research. They broadly overlap with the research priorities identified by the Palliative and end of life care Priority Setting Partnership (PeoPSP) in the UK, which used surveys and NGT to gain consensus from 1043 patients, carers and professionals [61]. It is to these themes that we now turn. We also propose a tentative action plan for each of these priority areas.

### Investigating issues about end of life decision making

Ethical issues around end of life decision making emerged as a key research priority. There is evidence that people with intellectual disabilities are not always sufficiently involved in end of life decision making, that assumptions are sometimes made about their quality of life and ability to cope with treatment, and that potentially life-saving treatments are not always offered because the person has intellectual disabilities [6, 62, 63]. The consequences are potentially extremely serious. They include, at worst, a risk of premature death; but there is also a risk of compromised quality of life if, for example, people are not offered treatments to ameliorate debilitating symptoms.

#### Possible future action

These issues are likely to be affected by national policies as well as cultural and attitudinal influences. They could usefully be researched in a range of countries with different approaches and practices. For example, exploring ways in which people with intellectual disabilities have been (or can be) meaningfully involved in advanced care planning and end of life decision making might be shared internationally and used to develop further guidance and materials.

### Mapping the scale and scope of the issue

At present, most official systems and services, including palliative care services, do not usually identify which of their service users have intellectual disabilities. They are, in effect, an invisible population and as such, there is a danger that their specific needs are overlooked, not seen as a priority or even potentially perceived as a problem [46]. In addition, by being unable to extract data at local, regional or national levels, we have little idea about the patterns of use (relative to the general population) of palliative care services for people with intellectual disabilities. The issue is confounded by a lack of insight into where people with intellectual disabilities currently live and die, and who is involved in their care and support at the end of life. There are likely to be huge variations across Europe, affected in part by variation in societal attitudes to disability and variations in palliative care provision for the general population. Mapping the scale and scope of the issue is a prerequisite for engendering the system and policy changes needed to ensure that all people with intellectual disabilities have the opportunity to access appropriate palliative care support and services that meet their specific needs.

#### Possible future action

In order to achieve this on a European level, international consensus on definitions is needed. An assessment of the influence of philosophies, laws and policies would also be helpful. Whilst it is of course possible to conduct studies focusing exclusively on issues around death, palliative care and intellectual disability, it may also be particularly useful to set up an intellectual disability strand in existing large-scale studies of the general population. This would have the advantage of making comparisons possible by highlighting similarities and differences between the palliative care needs of people with intellectual disabilities and those of the general population or other disadvantaged groups. One example of this is the Intellectual Disability Supplement to the Irish Longitudinal Study on Ageing [64].

### Investigating the quality of palliative care for people with intellectual disabilities

Research to date has highlighted unique challenges in achieving high quality palliative care for people with intellectual disabilities, as described at the start of this paper. There are real contextual differences between countries and cultures, but the extent to which this would influence models of best practice is uncertain. Investigating the quality of palliative care for people with intellectual disabilities in a wide range of European countries will enhance understanding of how good practice can be achieved, regardless of settings, circumstances or cultures. Researchers have started to investigate the perspectives and experiences of people with intellectual disabilities themselves, their families, care staff and professionals, but publications originate mostly from English-speaking countries.

#### Possible future action

The recent development of European-wide norms for palliative care of people with intellectual disabilities [65] may contribute to an increased awareness of the palliative care needs of this population and how these could be met. One important next step will be to assess how local and national agency and governmental policies align with the norms set out in the White Paper.

### Developing outcome measures and instruments for palliative care of people with intellectual disabilities

Unless there are outcome measures and instruments for palliative care that are appropriate and relevant to the circumstances of people with intellectual disabilities, it will not be possible to assess and compare the palliative care received by people with intellectual disabilities (between settings, regions and countries as well as in comparison with palliative care for the general population).

#### Possible future action

It may be possible to adapt one of the many current existing measures or indicators for assessing the quality of palliative care provision in the general population [66, 67]. We have not found any published work on this.

### European collaborations

It will be important to assess how the proposed research priorities align with the priorities of national agencies and governments across Europe (for example, with national palliative care research agendas, such as the UK Palliative and end of life care Priority Setting Partnership [61]) as this would give further leverage for obtaining research support. Careful consideration is then needed to determine which of the identified research themes (or sub-themes) need European-wide collaborations, and which specific research questions are best answered within national borders, given the wide variation in service provision and palliative care development [40]. Each of the four research priorities described above could usefully be addressed in a range of European countries and could benefit from international collaboration. Population studies benefit from greater numbers of participants; this is particularly salient in a field where numbers of people with intellectual disabilities within research studies might be too low for powerful statistical analysis. Analysis of palliative care provision across European borders, including aspects related to overall quality and to specific ethical issues and decision making, may highlight whether any barriers or issues are specific to local contexts, or whether they are experienced on a larger scale. This may help with the development of workable improvements. Research collaborations provide a powerful opportunity for sharing lessons learnt and thus enhancing practice.

## Conclusions

This paper describes the use of nominal group technique with a small group of international researchers and clinicians to propose themes for about a research agenda of palliative care for people with intellectual disabilities in Europe. This is a first step in the process of helping researchers to focus limited resources and research expertise on areas where it is most needed. It has also established an international community of support and exchange for research in intellectual disability, death, dying and palliative care. The next step is to cross-validated the proposed priorities with people with intellectual disabilities, their families and carers, clinicians, researchers and other stakeholders across Europe, and to then translate the research priorities into actual research studies by setting up European collaborations for specific studies that require such collaboration.

## Abbreviations

EAPC: European Association of Palliative Care
EoL: end of life
ID: intellectual disabilities
NGT: nominal group technique
PC: palliative care
